# Dumping Syndrome After Bariatric Surgery: Advanced Nutritional Perspectives and Integrated Pharmacological Management

**DOI:** 10.3390/nu17193123

**Published:** 2025-09-30

**Authors:** Raquel Cano, Daniel Rodríguez, Pablo Duran, Clímaco Cano, Diana Rojas-Gómez, Diego Rivera-Porras, Paola Barboza-González, Héctor Fuentes-Barría, Lissé Angarita, Arturo Boscan, Valmore Bermúdez

**Affiliations:** 1 Clínica General del Norte, Grupo de Estudio e Investigación en Salud, Barranquilla 080002, Colombia; raquelamiracano@gmail.com; 2 Endocrine and Metabolic Diseases Research Center, School of Medicine, University of Zulia, Maracaibo 4001, Venezuela; drdanielrodriguez.s18@gmail.com (D.R.); pabloduran1998@gmail.com (P.D.); antioxidante48@gmail.com (C.C.); 3 Escuela de Nutrición y Dietética, Facultad de Medicina, Universidad Andres Bello, Santiago 8370321, Chile; diana.rojas@unab.cl; 4 Universidad de la Costa, Departamento de Productividad e Innovación, Barranquilla 080001, Colombia; drivera23@cuc.edu.co; 5 Universidad Católica de la Santísima Concepción (Chile), Av. Alonso de Ribera 2850, Concepción 4330000, Chile; paola.barboza@ucsc.cl; 6 Vicerrectoría de Investigación e Innovación, Universidad Arturo Prat, Iquique 1110939, Chile; hefuentes_@unap.cl; 7 Escuela de Nutrición y Dietética, Facultad de Medicina, Universidad Andres Bello, Sede Concepción, Concepción 4260000, Chile; 8 Escuela de Medicina, Facultad de Medicina, Universidad del Zulia, Maracaibo 4001, Venezuela; arturojboscanmd@gmail.com; 9 Universidad Simón Bolívar, Facultad de Ciencias de la Salud, Centro de Investigaciones en Ciencias de la Vida, Barranquilla 080001, Colombia

**Keywords:** dumping syndrome, bariatric surgery, postprandial hypoglycaemia, gut hormones, obesity

## Abstract

Dumping Syndrome (DS) is a significant complication following bariatric surgery, particularly Roux-en-Y gastric bypass (RYGB). This condition is characterised by gastrointestinal and vasomotor symptoms resulting from altered anatomy and hormonal dysregulation, notably accelerated gastric emptying and an exaggerated release of gut peptides. Based on the timing of symptom onset after food ingestion, DS is classified as early (EDS) or late (LDS). The critical roles of peptides such as GLP-1, GIP, insulin, and YY peptide are highlighted, along with the involvement of neuroendocrine pathways in symptom manifestation. Diagnosis relies on a combination of clinical evaluation and dynamic testing, with the oral glucose tolerance test (OGTT) often considered a key reference standard for diagnosis. Initial management involves dietary modifications, emphasising the glycaemic index of foods and meal distribution. In cases where nutritional interventions are insufficient, pharmacotherapy with agents such as acarbose, somatostatin analogues (octreotide and pasireotide), GLP-1 receptor agonists (liraglutide), calcium channel blockers (verapamil), and emerging therapies, including herbal medicine, may be considered. For refractory cases, surgical options like bypass reversal or partial pancreatectomy are reserved, although their efficacy can be variable. Despite advancements in understanding and treating DS, further large-scale, randomised controlled trials are essential to validate novel strategies and optimise long-term management. This review provides an updated and comprehensive overview of the aetiology, pathophysiological mechanisms, diagnostic approaches, and current management strategies for DS.

## 1. Introduction

Bariatric surgery (BS), also referred to as “metabolic surgery”, has become one of the most commonly performed surgical procedures worldwide. This intervention, aimed at anatomically and functionally modifying various organs of the digestive system, seeks to induce biological changes beneficial to the individual’s health [1]. Numerous studies have demonstrated that BS is the most effective strategy for achieving and maintaining weight loss in individuals with severe obesity (SO) [2,3,4], and it has also been shown to reduce the risk of mortality from cardiovascular disease, cancer, and type 2 diabetes mellitus (T2DM) by up to 72% [5]. However, like any surgical procedure, BS is not without complications. These include gastrointestinal (GI) disorders such as gastric ulcers, thrombosis of the portal or mesenteric vein, abdominal pain, weight regain, and dumping syndrome (DS), the latter being one of the most prevalent complications in patients undergoing BS [6].

DS is a clinical condition characterised by rapid gastric emptying (RGE) and postprandial reactive hypoglycaemia (RPH), usually accompanied by vasomotor and GI symptoms [7]. Although its occurrence is more frequent following bariatric procedures [6,8], its aetiology may be multifactorial, including conditions such as T2DM, idiopathic diseases, and even intense physical activity [9,10,11]. From a pathophysiological perspective, DS involves a complex interplay of mechanical factors associated with the accelerated transit of osmotically active gastric contents into the small intestine, alterations of the enteric nervous system (ENS), and disruptions in the hormonal profile of the gastrointestinal tract (GIT) [6,7,8].

In this context, the clinical management of DS must be comprehensive and progressive. Dietary modification is the first-line treatment, followed by pharmacological interventions aimed at symptom control [12]. In refractory cases, surgical intervention may be considered; however, its outcomes are not always satisfactory and may entail new risks [6,7,13]. In response to these limitations, recent research has proposed emerging therapeutic alternatives, including somatostatin analogues (SAs), such as pasireotide and octreotide; glucagon-like peptide-1 (GLP-1) analogues, such as liraglutide; phytotherapy (PT); and calcium channel blockers, such as verapamil [14,15,16].

For these reasons, the objective of this review is to comprehensively examine the aetiology and pathophysiological mechanisms of dumping syndrome, as well as its diagnostic criteria and the most recent therapeutic advances, in order to contribute to scientific updates and the optimisation of care protocols for patients presenting with this condition.

## 2. The Aetiology of Dumping Syndrome

DS comprises a series of GI and vasomotor manifestations, which allow it to be classified into two types: early dumping syndrome (EDS) and late dumping syndrome (LDS) [17]. EDS occurs within the first hour after food intake and is accompanied by nausea, diarrhoea, borborygmi, tiredness, abdominal pain, and distension, along with vasomotor symptoms such as hypotension, palpitations, and fatigue, among others. LDS, also known as “post-bariatric hypoglycaemia” (PH), appears between 1 and 3 h after food intake and is characterised by typical hypoglycaemic symptoms such as generalised weakness, diaphoresis, light-headedness, dizziness, numbness of the lips, blurred vision, and confusion [18,19,20]. Although differences can be established between PH and LDS, such as the fact that PH occurs exclusively in the postprandial period and tends to manifest later than LDS, which typically appears within the first three months after RYGB [21], several authors propose unifying both conditions under the term RPH. This terminology would more accurately reflect the shared underlying pathophysiology of these metabolic disturbances [22]. In this context, the presentations of DS are mainly due to postoperative complications of BS [8]. However, less frequent causes have also been identified and may contribute to its development [10].

### 2.1. Bariatric Surgery: A Cornerstone in Dumping Syndrome Pathogenesis

BS comprises a set of surgical procedures aimed at inducing significant anatomical changes in the GIT. These modifications trigger a cascade of neurohormonal responses that promote weight loss and improve multiple metabolic parameters [20]. In this context, candidates for BS must meet specific established clinical criteria, notably a body mass index (BMI) of 40 kg/m^2^ or higher or a BMI between 35 and 40 kg/m^2^ accompanied by comorbidities such as T2DM, obstructive sleep apnoea, or an elevated cardiovascular risk. Additionally, individuals with a BMI < 35 kg/m^2^ may be considered in cases of poorly controlled T2DM [23]. Other requirements include the absence of clinically significant psychiatric disorders and undergoing a multidisciplinary assessment prior to the procedure [13].

Currently, six main types of surgical procedures are recognised within BS, classified according to their mechanism of action as restrictive, malabsorptive, or mixed. These procedures include jejunoileal bypass (JIB), Roux-en-Y gastric bypass (RYGB), vertical banded gastroplasty (VBG), biliopancreatic diversion (BPD) with or without duodenal switch (DS), adjustable gastric banding (AGB), and sleeve gastrectomy (SG) [24]. Restrictive techniques, such as SG, VBG, and AGB, work by reducing gastric volume to limit food intake. In contrast, procedures like BPD primarily aim to induce intestinal malabsorption, whereas interventions such as RYGB and BPD with duodenal switch (BPD/DS) have a mixed component, combining gastric restriction with intestinal malabsorption [13,25,26].

In this regard, RYGB has become the most common and preferred bariatric procedure worldwide due to its efficacy and safety profile [13,27]. However, a high prevalence of patients undergoing RYGB has been reported to experience symptoms consistent with DS, although, among these, only a small number develop a clinically significant form of the disorder. DS has been directly associated with RYGB, with the highest recurrence observed between 6 and 12 months following BS [28,29,30].

The pathophysiology of DS in these patients is linked to post-surgical anatomical changes that facilitate the rapid transfer of substantial volumes of undigested solid food into the small intestine. This accelerated transit may trigger both GI symptoms and metabolic disturbances. Moreover, these structural changes have been associated with alterations in the hormonal profile of the GIT, particularly in the secretion of incretins and other peptides that regulate appetite and glucose metabolism [13]. Vagal nerve (VN) damage has also been implicated as a contributing factor in the dysregulation of gastric emptying (GE) and autonomic intestinal control, potentially exacerbating the clinical manifestations of DS [13,29,30].

### 2.2. Alternative Aetiologies: Beyond Bariatric Surgery

Various clinical conditions have been associated with the pathophysiology of DS due to their link with rapid gastric emptying (RGE). These include idiopathic disorders, physical activity (PA), and, notably, T2DM, the latter being considered the most relevant non-surgical cause [15].

In this regard, studies conducted by Watson et al. [31] and Xie et al. [32] confirmed that GE is significantly faster in patients with T2DM compared to healthy individuals. RGE in individuals with T2DM can lead to the development of reactive hypoglycaemia (RH) due to counter-regulatory hormonal and neuroendocrine responses aimed at restoring normoglycemia [9]. Likewise, PA shows a causal relationship with RGE. Davis et al. [11] conducted a cross-sectional study involving 270 participants and found that individuals who engaged in regular physical exercise exhibited persistent RGE compared to sedentary subjects. This phenomenon may be attributed to increased levels of ghrelin, a gastrointestinal peptide with gastroprokinetic effects that stimulates GIT motility and promotes accelerated GE [33,34].

Moreover, various idiopathic conditions such as autonomic dysfunction, functional dyspepsia, and functional diarrhoea have also been associated with RGE [10]. This relationship is explained by alterations in the enteric nervous system, particularly in the myenteric plexus [35,36], which affect gastric accommodation and increase intragastric pressure. Additionally, increased contractions in the gastric body and antrum have been observed, accelerating the transit of contents toward the distal stomach [37]. These postprandial abnormalities in gastric motility and accommodation favour RGE, triggering a hypoglycaemic response characteristic of DS [38].

## 3. The Pathophysiological Landscape of Dumping Syndrome: From Mechanical Alterations to Hormonal Storms

DS involves various mechanical and biochemical mechanisms, largely originating from anatomical and functional changes in the GIT following BS. Therefore, understanding the normal digestive functioning before and after food intake is essential to elucidate the origin of DS.

In this context, the stomach functions as a flexible reservoir and pressure pump [39]. During ingestion, its proximal portion relaxes (gastric accommodation) through vagal stimuli, allowing gastric expansion and the intake of large volumes without an increase in intragastric pressure. Subsequently, tonic contractions of the gastric fundus propel the chyme towards the distal portion of the stomach [38,39]. And then through antroduodenal coordination, it is directed toward the pyloric canal and proximal duodenum [40,41,42].

Gastric activity is also regulated by neural stimuli, mediated by vagal afferents innervating sections of the GIT. These afferents project to the nucleus of the solitary tract (NTS) and are subsequently relayed to the dorsal motor nucleus of the vagus (DMV), which coordinates digestive motor responses [43]. Additionally, parasympathetic fibres and vagal afferents (VN) modulate gastric motor and hormonal responses via inhibitory (GIVC) and excitatory (GEVC) vagal gastric circuits [36,43,44,45].

In this regard, digestive processes are also mediated by GI hormones such as cholecystokinin (CCK), peptide YY (PYY), glucagon-like peptide-1 (GLP-1), and ghrelin. CCK exerts inhibitory effects on gastric secretion and motility, thereby delaying gastric emptying (GE) [46,47], and also acts on the VN, activating satiety-modulating signals [48,49]. Similarly, PYY slows down GE and distal intestinal transit along with GLP-1 [50] and can also inhibit intestinal motility and reduce food intake by acting on its Y2 receptors in the central nervous system (CNS) [51].

Other hormones relevant to the pathophysiology of DS include pancreatic polypeptide (PP), vasoactive intestinal peptide (VIP), neurotensin (NT), and motilin [12]. These regulate energy homeostasis and promote intestinal HCO_3_^−^ and Cl^−^ secretion, enhance GI motility, and stimulate biliary and pancreatic secretions [52,53,54,55]. GLP-1, however, acts via vagal afferents [56], stimulates insulin release [57,58], inhibits counter-regulatory hormones such as glucagon [59], and delays GE by stimulating nitrergic myenteric neurons, thereby reducing GI motility [60,61]. In contrast, ghrelin may accelerate GE through activation of its GHS receptors [45].

**Figure 1:**
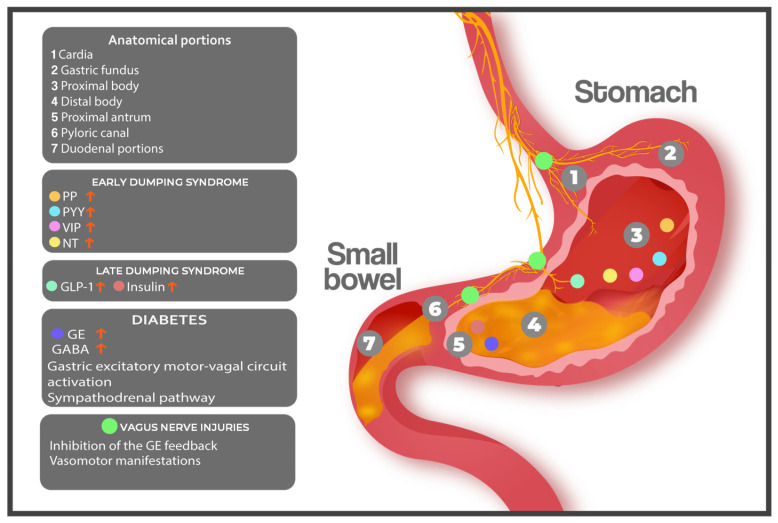
Pathophysiology of DS. After bariatric surgery, functional changes in the digestive system may lead to the development of dumping syndrome, which can be caused by an increase in the release of gastrointestinal hormones such as PP, PYY, VIP, NT, GLP-1, and insulin, or by vasomotor disorders resulting from alterations in the vagus nerve. **VN**: vagus nerve; **GE**: gastric emptying; **PP**: pancreatic polypeptide; **PYY**: peptide YY; **VIP**: vasoactive intestinal peptide; **NT**: neurotensin; **GLP-1**: glucagon-like peptide 1; **GABA**: gamma-aminobutyric acid; **LDS**: late dumping syndrome; **EDS**: early dumping syndrome.

Following BS, both the anatomy and physiology of the GIT are significantly altered, which may lead to the development of DS symptoms [62] (Figure 1). These changes include potential injury to the VN, disrupting neurohormonal signalling, and suppressing mechanical feedback involved in GE [15,63]. Additionally, the reduced gastric volume promotes accelerated nutrient delivery to the small intestine, triggering neurohormonal responses characteristic of DS due to impaired gastric relaxation, intragastric accommodation, early antral filling, and pyloric relaxation [6,8].

These hormonal alterations, along with enteric nervous system activation, contribute to the clinical manifestations of DS. In EDS, symptoms are associated with hypersecretion of HCO_3_^−^ and Cl^−^ and increased GI motility, leading to diarrhoea, nausea, vomiting, palpitations, headache, syncope, and abdominal cramps [6,7,8,12]. Later, accelerated intestinal transit promotes increased GLP-1 release and excessive insulin secretion, inducing postprandial RH with glucose levels <60 mg/dL, resulting in the typical symptoms of LDS [64,65].

Finally, another cause of GE dysfunction is diabetes. In these patients, recurrent hypoglycemia may result from increased glucose entry due to the loss of inhibitory hormonal action and activation of GABAergic neurons expressing GLUT-2 in the NTS, which in turn stimulates glucose uptake via the VN and the sympathoadrenal pathway [9]; however, despite advances in understanding the hormonal and neural mechanisms regulating GE, further clinical and preclinical studies are needed to clarify the diverse factors contributing to the onset of DS.

## 4. Diagnostic Approach to Dumping Syndrome: Clinical Criteria, Functional Testing, and Diagnostic Challenges

The broad range of non-specific manifestations of DS makes it difficult for healthcare professionals to arrive at a purely clinical diagnosis. For instance, abdominal cramps, bloating, and diarrhoea may be attributable to other complications of BS, such as stenosis, ischaemia, or fistula formation [13]. Likewise, gastro-oesophageal reflux (GOR) may be linked to the development of other conditions, such as T2DM, autonomic neuropathy, coeliac disease, or other gastrointestinal disorders, including inflammatory bowel disease, Crohn’s disease, and dyspepsia [38].

It is worth noting that, in some cases, differentiating between diabetic gastroparesis and gastroparesis proves difficult due to the significant similarity in their clinical presentations. However, the frequency of diarrhoea and abdominal pain may be particularly useful in clarifying which of the two pathologies is present. In addition, other differential diagnoses include idiopathic diarrhoea, pancreatic insufficiency, and lactose intolerance [14,15,17]. Along the same lines, hypoglycaemia is another of the most common complications following BS, and it may occur concurrently with DS or independently, giving rise to various phenomena related to this alteration, such as nesidioblastosis, insulinomas, or adverse effects from the use of antidiabetic medications [6,7,12,66,67].

As a result, several more specific diagnostic tools and methods have emerged to aid in identifying patients with this syndrome [6]. First, the Sigstad scoring system is a diagnostic tool that helps determine the presence of DS following oral glucose administration [68,69]. In this scale, the presence of ≥7 symptoms suggests a high probability of DS, whereas scores <4 indicate the need to consider other aetiological alternatives. Additionally, the scale allows for the classification of the type of DS according to the timing of symptom onset, diagnosing EDS if symptoms occur within <1 h and LDS if after >1 h [28,70]. However, there is no substantial evidence supporting the efficacy of this scale for diagnosing DS [19].

However, the Arts scale focuses on assessing both symptom severity and the type of DS present in patients, using a 4-point Likert-type scale based on the intensity of symptoms experienced either within the first 60 min or after the first hour post-ingestion (EDS and LDS, respectively). Items are rated on a scale from 0 to 3, where 0 denotes absence of symptoms, 1 denotes mild intensity, 2 is moderate, and 3 is severe intensity [71,72].

Additionally, confirmatory diagnostic tests have been developed, such as the assessment of GI motility using magnetic resonance imaging (MRI) and GE scintigraphy. The latter involves the ingestion of a small meal containing a radioactive tracer, allowing for the measurement of GE rate over 1 to 4 h post-ingestion; however, these tests have low sensitivity and specificity for diagnosing DS [6,7].

More recently, the Mixed Meal Tolerance Test (MMTT) has gained relevance as a functional tool to evaluate both EDS and LDS, offering a more physiological simulation of the postprandial response compared to the traditional oral glucose tolerance test (OGTT) [6]. The MMTT involves the ingestion of a standardised meal containing a balanced mixture of carbohydrates, proteins, and fats—typically lower in carbohydrate content than the OGTT—to avoid artificially high glycaemic and insulin peaks. One commonly used formulation consists of 241 kcal, with 15.5 g protein, 10 g fat, 20.7 g carbohydrates, and 3.1 g fibre, aligning with nutritional recommendations following BS [73].

This test allows for the simultaneous evaluation of glycaemic excursions, insulin secretion, incretin response, and the presence of hypoglycaemia, providing a comprehensive metabolic and hormonal profile associated with DS symptoms. In EDS, physiological changes such as a haematocrit increase >3% (due to splanchnic fluid shift) and heart rate elevation >10 beats per minute are considered indicative, while in LDS, a plasma glucose drop <70 mg/dL, particularly <54 mg/dL, is often used as a threshold for hypoglycaemia. However, clinical findings suggest limited diagnostic performance: in a cohort of 56 patients with self-reported DS symptoms, only 28% had a positive Sigstad Score (≥7) during MMTT, and neither a haematocrit increase >3%, a heart rate elevation >10 bpm, nor glucose drops <70 mg/dL were reliably associated with symptom onset [73,74].

In clinical practice, the MMTT may be used in conjunction with symptom-based scales such as the Sigstad or Arts scales, enhancing the diagnostic accuracy by correlating objective metabolic changes with subjective symptomatology. Its application is particularly valuable in research settings and specialised centres, where precise temporal correlation between ingestion, symptoms, and biochemical markers is required for an accurate diagnosis.

## 5. Therapeutic Approach to Post-Bariatric Dumping Syndrome: From Nutrition to Pharmacotherapy

Since its discovery, DS has been recognised as a postoperative complication characterised by a wide range of clinical manifestations, often difficult to manage. To address this therapeutic challenge, various strategies have been developed, ranging from surgical procedures to modify GI anatomy to specialised nutritional interventions and pharmacological options aimed at modulating hormonal responses and intestinal motility.

The management of post-bariatric DS requires a stepped and individualised therapeutic approach, with nutritional interventions forming the cornerstone and first line of treatment. This approach is not merely symptomatic but seeks to modulate the profound physiological changes induced by bariatric procedures, particularly those affecting GE and the entero-hormonal response [6,7].

### 5.1. Pathophysiological Principles Guiding Nutritional Intervention

Nutritional strategies in DS aim to modulate key pathophysiological mechanisms, including the rate of GE, the osmotic load delivered to the small intestine, and the subsequent neurohormonal responses that underlie EDS and LDS symptoms [7]. However, the clinical presentation of DS can be broadly categorised into early and late forms, each with distinct physiological triggers and symptom profiles. Therefore, tailoring dietary interventions based on the predominant type—EDS or LDS—enhances therapeutic precision and symptom control.

#### 5.1.1. Nutritional Intervention for Early Dumping Syndrome

EDS typically occurs within 30–60 min after food intake and is primarily mediated by rapid GE, resulting in sudden osmotic shifts, luminal distension, and release of vasoactive peptides. The following interventions are recommended:

Small and Frequent Meals. Consuming 5 to 6 small meals per day reduces the volume of chyme entering the small intestine at any one time, thereby attenuating osmotic load and neural-hormonal responses. A large bolus in the proximal intestine triggers fluid shifts from the intravascular space into the lumen, leading to hypovolemia-related symptoms such as dizziness, tachycardia, and weakness [12]. Smaller meal volumes help minimise these effects and reduce symptom severity. Additionally, patients are advised to eat slowly and chew thoroughly, as this behavioural measure can further delay GE and limit rapid intestinal exposure to undigested food, contributing to symptom control in EDS [74].

Separation of Solids and Liquids. Liquids empty faster than solids, especially in post-surgical gastric anatomy with altered pyloric control or anastomotic enlargement [75]. This rapid transit enhances osmotic load and intestinal distension. Patients are advised to drink fluids at least 30 min before or after solid meals to slow overall GE and reduce early symptoms [74].

Moderation of Dietary Fat. While fat can delay GE, excessive intake may exacerbate early symptoms in some individuals due to delayed but large-volume intestinal delivery, triggering distension and neuroendocrine release [76]. Therefore, moderate fat intake is advised, especially in the EDS context.

Lying Down After Meals. For patients who are unable to adhere to initial dietary modifications or in whom first-line strategies have proven ineffective, lying down for approximately 30 min after meals may serve as a supportive behavioural measure. This position can help slow GE and mitigate hypovolemia-related symptoms such as dizziness, fatigue, and tachycardia, offering additional relief in EDS [74].

#### 5.1.2. Nutritional Intervention for Late Dumping Syndrome

LDS typically occurs 1–3 h after meals and is characterised by RH secondary to rapid glucose absorption and exaggerated insulin release. The primary focus is to stabilise glycaemic responses:

Low glycaemic index carbohydrates, as opposed to rapidly absorbed high-GI carbohydrates (e.g., refined sugars and white bread), lead to postprandial hyperglycaemia followed by a sharp insulin spike and subsequent hypoglycaemia [9,16]. Replacing them with complex, low-GI carbohydrates (e.g., whole grains and legumes) slows glucose absorption, mitigating these fluctuations [77].

Increased soluble fibre intake: Soluble fibres, such as pectin, guar gum, and glucomannan, form viscous gels in the intestinal lumen, slowing gastric and intestinal transit and blunting postprandial glucose peaks [78]. This leads to more stable glycaemia and reduces LDS [74,79]. However, tolerance to fibre supplements varies; some patients may experience bloating and flatulence due to fermentation in the colon [74].

Adequate Protein and Fat. Protein and fat slow GE and glucose absorption, contributing to satiety and glycaemic control [7,76]. Although fat must be moderated in EDS, it plays a protective role in LDS by delaying glucose entry into the small intestine [80].

Among the various dietary modifications. The most foundational and consistently recommended strategies are the consumption of small and frequent meals, separation of liquids and solids, and the use of low glycaemic index carbohydrates. These interventions directly target the core pathophysiological mechanisms of both EDS and LDS and form the basis of nutritional therapy. Along with increased soluble fibre intake, eating slowly and chewing thoroughly, and postprandial recumbency when needed, all these measures are supported by clinical experience and observational data, carrying a Level of Evidence of III and a Grade of Recommendation: B [74]. While no single strategy universally prevails, starting with meal size and composition adjustments is often the most impactful and practical first step.

### 5.2. Fundamental Dietary Strategies and Specific Considerations

The basic dietary recommendations for managing DS, derived from the pathophysiological principles described above, focus on modifying meal frequency, volume, and composition, as well as the eating pattern. Consuming 5–6 small meals throughout the day is essential, as avoiding large volumes in a single sitting is beneficial [6]. The volume of each meal should be adapted to individual tolerance and the type of bariatric procedure performed. Separation of fluid intake after meals is critical to avoid dilution of chyme and accelerated GE of solids [6,7]. Although this recommendation is based on the pathophysiology of DS and digestive physiology, there is little clinical evidence, and more clinical studies are needed to assess the effect of fluid intake on DS. In terms of macronutrient composition, protein intake (lean sources such as chicken, fish, and legumes) and healthy fats (avocado, nuts, and olive oil) should be prioritised at every meal to promote prolonged satiety and better glycaemic control.

It is imperative to drastically limit simple and refined carbohydrates (high glycaemic index carbohydrates: sweets, sugary drinks, white bread, and non-wholegrain pasta) and opt instead for complex or low glycaemic index carbohydrates rich in fibre (vegetables, fruits with skin, and whole grains) [6,7]. Increasing consumption of foods naturally high in soluble fibre is beneficial, and soluble fibre supplements (such as psyllium or guar gum) may be considered cautiously—starting with low doses and adjusting according to tolerance to minimise gastrointestinal side effects [74].

The pace of eating is equally important; eating slowly and chewing food thoroughly facilitates mechanical digestion in the gastric pouch and reduces the size of food particles entering the intestine, potentially aiding more controlled emptying [6]. Additionally, lying down for 20–30 min after meals may help delay GE via gravitational effects and mitigate early vasomotor symptoms by reducing fluid shifts [6].

The inclusion of fats in the diet has generated some controversy. Although fats physiologically delay GE, consuming large quantities or combining them with simple carbohydrates may paradoxically exacerbate DS symptoms in some patients [76]. This phenomenon may relate to the release of intestinal peptides or the osmotic load of fat digestion products. Therefore, an individualised evaluation of fat tolerance and distribution throughout the day is advised.

Ensuring adequate hydration and maintaining electrolyte balance is crucial, especially for patients experiencing vomiting and diarrhoea, including the administration of clear fluids and electrolyte solutions to prevent dehydration [81]. Proper management of sodium and potassium content is particularly crucial in maintaining fluid and electrolyte balance across the body’s compartments. Oral rehydration solutions may serve as an effective rehydration therapy once such symptoms present [21].

To prevent dehydration effectively, patients should be advised to consume fluids in small amounts throughout the day [6,7], as previously mentioned, avoiding fluid intake with meals. It is essential to consume fluids 30 to 60 min after main meals. The total daily water intake should range between 1.8 and 2 litres. It is crucial to maintain a slow drinking pace by using small sips and avoiding the use of drinking aids such as straws [81]. Lastly, consuming fluids at a lukewarm temperature is a key recommendation in DS management [6].

### 5.3. Towards Precision Nutrition: Individualisation and Advanced Tools

The heterogeneity in the presentation of DS and response to nutritional interventions underscores the need for a highly individualised approach that goes beyond general recommendations and is supported by precise assessment tools. A comprehensive nutritional evaluation is the starting point, including not only general dietary habits but also the specific identification of foods or meal patterns perceived by the patient as symptom triggers, the frequency and severity of these symptoms, and a thorough assessment of nutritional status to detect possible micronutrient deficiencies, which are common among post-bariatric patients [17].

The systematic use of a diary in which the patient records their food intake in detail (type, quantity, and composition), along with the onset, type, and severity of postprandial symptoms, is an invaluable tool. This record enables the identification of specific food intolerance patterns, the objective correlation of intake with symptom onset, and empirical, personalised adjustments to recommendations [17]. In complex cases where trigger identification is difficult, or the response to initial recommendations is limited, controlled food tolerance testing in a clinical setting may provide objective information. These tests involve the controlled administration of foods or solutions with varying macronutrient loads and the monitoring of symptomatic and, where possible, metabolic responses (e.g., blood glucose levels and hormone levels) [74].

Continuous glucose monitoring (CGM) emerges as a cutting-edge tool for individualising nutritional management, particularly in late-stage diabetes with reactive hypoglycaemia. By providing real-time glycaemic profiles in response to food intake, CGM allows precise identification of foods, food combinations, and meal patterns that induce significant glycaemic excursions (peaks of hyperglycaemia followed by hypoglycaemic dips). This detailed information facilitates dietary adjustments based on objective data, allowing immediate feedback to the patient and optimisation of the nutritional plan to minimise hypoglycaemic episodes [82].

### 5.4. The Gut Microbiota Axis and Its Nutritional Modulation

Growing evidence on the role of post-bariatric gut dysbiosis in the pathophysiology of DS opens new avenues for nutritional management, focusing on the modulation of the microbial ecosystem [83]. Diet is a key determinant of gut microbiota composition and function, and nutritional interventions can be designed to influence this axis, as discussed in a later chapter. Modulating the microbiota through diet—particularly through the intake of fibre and complex carbohydrates—directly affects the availability of substrates for colonic bacterial fermentation, influencing the production of short-chain fatty acids (SCFAs) [79]. SCFAs such as butyrate, propionate, and acetate are crucial for intestinal epithelial health barrier function and can affect the secretion of gut peptides such as GLP-1 and PYY, thereby modulating motility and glucose metabolism [84].

The strategic inclusion of foods rich in prebiotics (e.g., inulin and fructo-oligosaccharides found in onions, garlic, leeks, asparagus, and resistant starch in green bananas, legumes, and cooked and cooled potatoes) may selectively promote the growth of beneficial bacteria associated with favourable metabolic outcomes, such as *Akkermansia muciniphila* and *Faecalibacterium prausnitzii* [85]. These bacteria can positively influence gut barrier function, reduce low-grade inflammation, and modulate SCFA production and hormone secretion.

Although direct evidence on the impact of specific probiotics on DS symptoms is still limited and requires further research, modulation of the microbiota through probiotics (supplements with specific strains) or fermented foods (live-culture yoghurt, kefir, and sauerkraut) could theoretically have a positive influence on microbial composition, gut motility, low-grade inflammation, and hormonal response in some patients [86,87]. Selecting specific probiotic strains with documented effects on motility or intestinal barrier function could be relevant in the future.

FODMAPs (fermentable oligosaccharides, disaccharides, monosaccharides, and polyols) are poorly absorbed carbohydrates that can provoke GI symptoms such as bloating, abdominal pain, and diarrhoea [88]. A low-FODMAP diet has proven effective in alleviating these symptoms, particularly in patients with functional digestive disorders such as irritable bowel syndrome [89,90]. Implementing a low-FODMAP diet may be considered for DS patients presenting with these symptoms. This strategy, always under the supervision of a specialist dietitian-nutritionist, aims to reduce the fermentable carbohydrate load and evaluate its impact on the patient’s symptomatology.

### 5.5. Transition to Pharmacotherapy

When nutrition alone is insufficient—despite the rigorous implementation of optimised and individualised nutritional strategies, which constitute the first-line treatment and are adequate for managing symptoms in the majority of patients [6,8,91]—a subgroup of post-bariatric patients with DS will continue to experience significant symptoms that considerably impact their quality of life. The prevalence of dumping symptoms varies depending on the type of bariatric procedure, being reported in approximately 40% to 75% of patients who have undergone RYGB and around 15.6% to 40% following SG [92,93].

Although many mild cases resolve over time and with dietary modification, meta-analyses and systematic reviews indicate that a notable subgroup, estimated to be between 1% and 10%, experiences severe and persistent symptoms that do not adequately respond to dietary measures and, therefore, require additional management [94]. In these cases—refractory to nutritional therapy despite adherence and careful adjustment of recommendations—therapeutic escalation is justified. Pharmacological interventions should then be considered to modulate the pathophysiological mechanisms that are not fully controlled by diet, such as accelerated GE, exaggerated hormonal release, or RH. Thus, pharmacotherapy is positioned as the second-line treatment in the comprehensive management of DS, complementing nutritional measures and providing symptomatic relief when these prove insufficient.

### 5.6. Pharmacotherapy Options: Mechanisms and Evidence

In decisions regarding pharmacotherapeutic escalation, various compounds have been studied over the years to achieve the desired therapeutic outcomes for this condition, including acarbose, diazoxide, and somatostatin analogues (SAs) such as octreotide and pasireotide [6]. However, recent studies have increasingly focused on the use of GLP-1 receptor analogues and motilin hormones (MHs) as potential adjunct treatments, and calcium channel blockers, an established strategy, have also been explored as part of the therapeutic options for managing DS.

In this regard, acarbose is an inhibitor of the enzyme alpha-glucosidase hydrolase, and its mechanism of action involves slowing the absorption of carbohydrates in the small intestine. Additionally, it inhibits the synthesis of monosaccharides from carbohydrates, promoting more appropriate insulin release and thus playing a significant role in the treatment of T2DM. Nonetheless, this drug is not free from adverse effects, which include bloating, flatulence, and diarrhoea. Its administration has been shown to improve symptoms of RH in post-BS patients [95,96]. In this context, Ritz et al. [97], in a prospective study of patients diagnosed with LDS following SG, demonstrated that administering 50–100 mg of acarbose three times daily improved symptoms in 87.5% of patients. However, despite the dietary plan and pharmacological protocol implemented, complete normalisation of glycaemic levels characteristic of DS was not achieved.

Diazoxide, a non-diuretic derivative of benzothiadiazine, is used in the treatment of hyperinsulinaemic hypoglycaemia due to its ability to bind to ATP-sensitive potassium channels on pancreatic β-cells, causing cellular hyperpolarisation, leading to inhibition of insulin secretion [98,99]. In this context, Thondam et al. [100] assessed the efficacy of 50 mg of diazoxide administered twice daily in three bariatric patients diagnosed with T2DM, showing improvement in hypoglycaemic symptoms in all cases. Similar findings were reported by Mejía et al. [101], suggesting that this drug may represent an effective alternative for treating DS-associated hypoglycaemia.

Regarding SAs, octreotide is a synthetic peptide analogue of somatostatin that has effects, including delaying GE by modulating the migrating motor complex and inhibiting the synthesis of intestinal peptides, vasoactive substances, fluids, and electrolytes—all of which are implicated in diabetic gastroparesis pathophysiology [7]. Sato et al. [102] evaluated the efficacy of octreotide in a case study involving a 47-year-old woman who had undergone BS and presented with symptoms consistent with diabetic symptoms, observing suppression of GLP-1 and GIP concentrations, along with reductions in insulin, C-peptide, and plasma glucose levels. Therefore, the authors concluded that octreotide is an effective therapeutic measure in the treatment of EDS.

Similarly, pasireotide, another SAs, can bind to four of the five subtypes of somatostatin receptors, allowing it to regulate blood glucose levels by inhibiting both glucagon and insulin secretion. It also inhibits the release of GLP-1 and PYY, key hormones in the pathophysiology of DS [103,104]. Additionally, new therapeutic tools have been proposed, representing progress in the treatment of DS protocols. Among them, liraglutide—a GLP-1 analogue—stimulates insulin release, inhibits glucagon secretion, stabilises plasma glucose levels, reduces GE rate, and increases satiety. Although it offers clinical benefits, it may also induce undesirable GI side effects such as nausea, diarrhoea, constipation, and vomiting [105,106]. In this respect, a case report involving a 52-year-old woman with DS symptoms after BS found that administration of 0.6 mg/day of liraglutide reduced insulin peaks during an OGTT [107]. Consistently, a clinical study involving 27 post-BS patients with LDS treated with GLP-1 analogues reported that 54% experienced a reduction in the frequency and intensity of hypoglycaemic episodes, while 46% presented with no episodes [108]. Despite these findings, further clinical studies are needed to support the use of liraglutide in the treatment of DS.

Numerous studies are currently underway to explore the use of motilin hormones (MHs) in clinical practice. Their utility in regulating GI disorders has been demonstrated through their role in the synthesis and release of hormones acting on the GIT [87]. In this vein, Hong et al. [15] reported significant improvements in GI symptoms associated with DS, as observed in a meta-analysis of three clinical trials involving a total of 174 patients, compared to those observed with conventional pharmacological treatments. Nevertheless, further studies are required to evaluate the efficacy of MHs in this therapeutic context.

Finally, calcium channel blockers have been studied as therapeutic alternatives for EDS due to their ability to regulate glycaemic concentrations. Among these, verapamil, a calcium channel blocker, can inhibit insulin release by preventing calcium ion entry into pancreatic β-cells, which may contribute to reducing hypoglycaemic symptoms in patients with post-bariatric hypoglycaemia (PBH). Moreira et al. [109], in a case report of a 26-year-old female patient experiencing frequent hypoglycaemia episodes, examined the use of verapamil at a dose of 80 mg twice daily, observing a reduction in both frequency and intensity of symptoms. However, other studies suggest that verapamil does not have significant effects in the treatment of PBH [110].

### 5.7. Revisiting Surgical Alternatives in Refractory Cases

Due to the variety of surgical techniques currently employed in bariatric practice, several studies have sought to identify the most effective surgical approaches for relieving symptoms of DS, particularly in cases where nutritional and pharmacological interventions fail. Among the procedures considered are gastric pouch restriction (GPR), Roux-en-Y gastric bypass reversal (RYGBR), and pancreatic resection (PR) [6].

GPR involves reducing the size of the gastric pouch created during the initial RYGB procedure to restore a more restrictive gastric capacity and delay GE [111]. RYGBR, however, entails anatomical reversal of the original bypass, including detachment of the gastrojejunostomy, re-establishment of gastric continuity via the greater curvature, and restoration of normal jejunal flow [112,113]. PR consists of segmental resection of the pancreas—occasionally extending to the common bile duct—and has been proposed in rare and extreme cases of intractable postprandial hypoglycaemia [114].

An evaluation of 14 studies involving 75 patients with PBH revealed symptomatic improvement in 82% of patients following GPR, 76% following RYGBR, and 67% following PR [115]. However, PR is generally regarded as a high-risk, last-line intervention due to its association with serious complications, including recurrent or paradoxical hypoglycaemia and pancreatic insufficiency [116].

Importantly, emerging evidence has drawn attention to a potential pathophysiological link between partial small bowel obstruction and intractable postprandial hypoglycaemia, often accompanied by recurrent abdominal pain or discomfort. In a large population-based study, 1429 patients were surveyed a median of 4.7 years after undergoing RYGB and found that 88.6% reported at least one symptom, with 34.2% experiencing abdominal pain leading to healthcare contact and 29.1% requiring hospitalisation [117]. Although this study did not directly evaluate the mechanical causes of these symptoms, the high prevalence of post-RYGB abdominal pain suggests that anatomical or functional abnormalities, such as intermittent bowel kinking or partial obstruction, may contribute to persistent symptoms, including refractory hypoglycaemia. In line with this, Laurenius et al. described a series of patients with severe PBH and postprandial abdominal discomfort who underwent surgery to correct partial small bowel obstruction (e.g., adhesions or dysfunctional anastomosis). Among the 21 patients using hypoglycaemia medications preoperatively, 90.5% discontinued treatment after surgery. Of the total 80 patients interviewed, 8% became entirely free of hypoglycaemic symptoms, while 71% reported significant improvement, supporting a potential association between severe PBH and partial small bowel obstruction [118]. Furthermore, mechanistic studies have demonstrated that bile diversion and altered small bowel anatomy after RYGB can influence glucose absorption by modulating sodium-dependent glucose transporters, supporting the idea that anatomic modifications may play a direct role in postprandial glycaemic dysregulation [119].

Taken together, these findings highlight the importance of considering subtle mechanical causes—such as kinking, strictures, adhesions, or partial obstruction—as potential contributors to intractable symptoms in DS. Identifying such abnormalities, through imaging or exploratory surgery, may guide more effective and targeted interventions. Given the complexity and potential risks, surgical reintervention should remain a last-resort therapeutic strategy, reserved for carefully selected patients in whom anatomical abnormalities are suspected and conservative management has failed.

## 6. Modulation of Gut Microbiota and Micronutrient Balance in Post-Bariatric Patients: Emerging Links with Dumping Syndrome and Therapeutic Perspectives

Bariatric procedures, such as RYGB and SG, induce profound anatomical and physiological changes in the GIT. These changes are reflected not only in digestive dynamics and nutrient absorption but also in the intestinal ecology. One emerging phenomenon in this context is postoperative intestinal dysbiosis, understood as an imbalance in the composition, diversity, and function of the gut microbiome, which may play a modulatory role in the development or exacerbation of DS [120].

Prior to BS, the gut microbiota of individuals with severe obesity is typically characterised by a high proportion of the Firmicutes phylum, including families such as Lachnospiraceae and Clostridiaceae, and genera such as *Coprococcus* and *Lactococcus*, which are associated with increased energy extraction capacity and an unfavourable metabolic profile [121]. A significant abundance of *Blautia* and *Ruminococcus gnavus*, species linked to chronic inflammation and insulin resistance, is also observed [122].

Following surgery, there is a marked reconfiguration of the intestinal microbial ecosystem. Bacterial diversity increases, and there is an observed rise in the abundance of the Bacteroidetes and Proteobacteria phyla, particularly the Enterobacteriaceae family [123]. Similar alterations have been reported, including an increase in Proteobacteria and Verrucomicrobia and a reduction in Firmicutes, along with a decrease in overall microbial diversity [124,125]. Some studies have specifically identified, following RYGB and SG, an increase in *Escherichia coli* and a reduction in beneficial genera such as *Lactobacillus* and *Bifidobacterium* [126,127,128]. At the genus level, there is an increase in *Bacteroides*, *Parabacteroides*, and *Slackia*, while species such as *Bacteroides thetaiotaomicron* and *Akkermansia muciniphila* become more prevalent—the latter being associated with improvements in glucose homeostasis and lipid metabolism [129,130,131].

These modifications are associated with multiple relevant pathophysiological effects: increased intestinal permeability, activation of low-grade inflammatory responses, altered production of metabolites such as short-chain fatty acids (SCFAs), and dysfunction in the secretion of gut hormones, including GLP-1, PYY, and ghrelin—all involved in the regulation of gastric motility and emptying [83,132,133]. Concurrently, a decrease in genera such as *Coprococcus* and *Lactococcus* may reflect reduced butyrate fermentation and lower systemic inflammation [121].

Interindividual variability in the composition and response of the gut microbiota following BS is a well-documented phenomenon of growing clinical interest. Several studies have shown that, although general patterns of change in the postoperative microbiota exist—such as an increase in certain bacterial genera and a decrease in others—the magnitude and direction of these changes can vary significantly among individuals. For instance, one study found that the relative abundance of *Akkermansia muciniphila* was correlated with remission of T2DM in some patients but not in others, suggesting a personalised microbial response to surgery [134].

It is essential to consider the individuality of the microbial response when planning postoperative therapeutic strategies, including nutritional interventions and the use of probiotics or prebiotics, in order to optimise clinical outcomes and minimise associated complications. In DS—where symptoms result from accelerated GE and secondary intestinal hypersecretion—these alterations could act as an amplifying factor. For example, dysbiotic microbiota may increase rapid colonic fermentation of high-glycaemic-index carbohydrates, exacerbating gas production and intestinal distension in EDS. Similarly, the interaction between dysbiosis and carbohydrate metabolism may intensify the postprandial hyperglycaemic peaks followed by RH characteristic of LDS [82].

**Figure 2:**
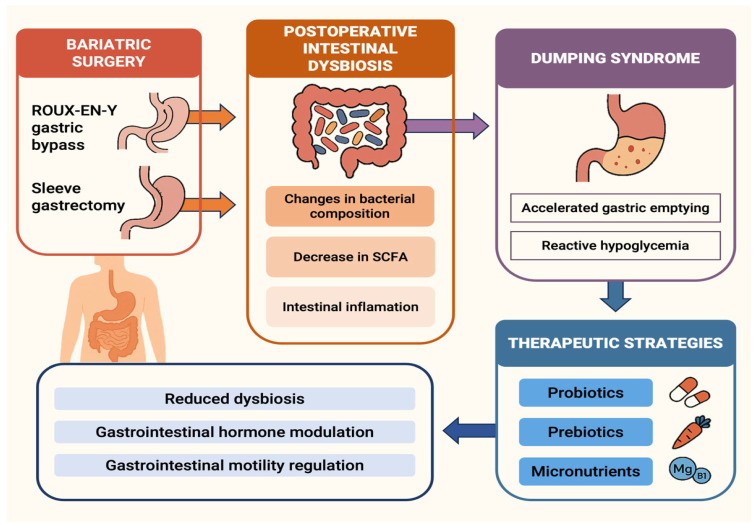
Postoperative intestinal dysbiosis as a potential modulator of dumping syndrome after bariatric surgery and its therapeutic implications. Bariatric procedures such as Roux-en-Y gastric bypass (RYGB) and sleeve gastrectomy induce profound anatomical and physiological changes that extend beyond nutrient absorption, also affecting the composition and function of the gut microbiota. These changes often result in postoperative intestinal dysbiosis, characterised by reduced microbial diversity, altered production of short-chain fatty acids (SCFAs), and low-grade intestinal inflammation. Such dysbiosis may contribute to or exacerbate dumping syndrome (DS) by promoting rapid colonic fermentation of high-glycaemic carbohydrates, increasing intestinal gas production, and disrupting glucose homeostasis—culminating in both early and late dumping symptoms. Therapeutic strategies aimed at restoring microbial balance through probiotics, prebiotics, and targeted micronutrient supplementation (e.g., magnesium and thiamine) have shown potential in modulating gut motility, hormone secretion, and metabolic outcomes. While evidence is still emerging, these microbiota-directed interventions represent a promising avenue for managing DS in the post-bariatric population. **SCFA**: short-chain fatty acids.

Traditional management of DS has included dietary, pharmacological, and even secondary surgical modifications. However, strategies aimed at modulating the gut microbiota could emerge as a novel therapeutic avenue (Figure 2). Various studies have shown that the administration of probiotics, prebiotics, and synbiotics can restore microbial diversity, reduce inflammation, and improve glucose metabolism in obese or T2DM patients—conditions that share physiological alterations with post-bariatric individuals in whom DS is highly prevalent [135,136,137]. Specifically, *Lactobacillus* and *Bifidobacterium* strains have been associated with improvements in intestinal barrier integrity and reductions in metabolic endotoxaemia [86].

The clinical trial conducted by Wagner et al. [138] demonstrated the positive effect of probiotic supplementation based on *Lactobacillus acidophilus* and *Bifidobacterium lactis* (5 billion CFU/strain) following RYGB. Although the prevalence of small intestinal bacterial overgrowth (SIBO) and the mean scores on the Gastric Symptom Rating Scale (GSRS) remained similar to the control groups over time, patients receiving probiotics reported an immediate improvement in certain GI symptoms such as bloating, as well as a reduction in abdominal pain 90 days after surgery. These findings may vary over time, as shown by Melali et al. [139], where symbiotic supplementation (probiotics and prebiotics) based on a strain mix, including *Lactobacillus rhamnosus*, *Lactobacillus casei*, *Lactobacillus bulgaricus*, *Lactobacillus acidophilus*, *Bifidobacterium breve*, *Bifidobacterium longum,* and *Streptococcus thermophilus* (1 billion CFU/strain), together with fructooligosaccharides (FOSs), was associated with statistically superior GI quality of life index (GIQLI) scores compared to control groups both before and 6 months after surgery (*p* < 0.001 for probiotics and *p* = 0.03 for placebo). The need for randomised clinical trials specifically addressing post-bariatric DS, supported by robust methodology, could be crucial in positioning probiotic supplementation as an adjunctive measure for these patients.

In a recent clinical trial, it was demonstrated that administering probiotics over six months after RYGB supported sustained weight loss and improved fasting glycaemia and insulin sensitivity, suggesting a positive interaction between the microbiota and postoperative metabolism [140]. Furthermore, metagenomic studies have shown that certain gut bacterial profiles—such as higher abundances of *Akkermansia muciniphila* or *Faecalibacterium prausnitzii*—may be linked to better glycaemic control and greater postprandial stability, which would be desirable in patients experiencing LDS [85]. This effect raises the possibility of using these bacteria as prognostic markers or even as targeted therapeutic agents. A four-year metagenomic study before and after BS demonstrated alterations in the composition of the gut microbiota—specifically, increases in *Proteobacteria* and *Clostridia*—associated with obesity remission, dietary energy extraction, and GI symptoms such as diarrhoea and malabsorption, which are common in DS [121]. This research also highlighted a significant reduction in SCFAs, such as acetate, propionate, and butyrate, showing a negative correlation with the genera *Butyricimonas* and *Parabacteroides*; the latter has been inversely correlated with prolonged post-surgical insulin concentrations, potentially implicated in the reduction in RH in LDS.

Beyond interventions involving probiotics and prebiotics, supplementation with specific micronutrients has been proposed as a promising strategy to modulate the gut microbiota and improve metabolic outcomes in post-bariatric patients. Thiamine is essential for carbohydrate metabolism, and its deficiency can adversely affect GI function. Following BS, particularly in malabsorptive procedures, patients are at increased risk of thiamine deficiency, which may contribute to symptoms such as nausea, vomiting, fatigue, and Wernicke’s encephalopathy in severe cases [141,142]. A recent study, currently available as a preprint and pending peer review, identified a positive correlation between the abundance of a *Coprococcus* species associated with magnesium and thiamine intake and (BMI in patients who underwent malabsorptive BS [143]. Trials have demonstrated that thiamine supplementation can modulate the gut microbiota, thereby promoting a favourable microbial balance. For example, a study in mice fed a high-fat and high-fructose diet found that thiamine administration increased the abundance of beneficial bacteria, such as *Bifidobacterium pseudolongum*, and reduced the abundance of pro-inflammatory bacteria, such as *Ruminococcus gnavus*. These changes were associated with improved intestinal barrier function and decreased endotoxaemia, suggesting an anti-inflammatory effect beneficial for glucose metabolism [144]. Moreover, SIBO has been proposed to induce thiamine deficiency by altering the intestinal ecosystem, highlighting the importance of promoting a balanced microbiota [145]. In line with these findings, the potential of randomised clinical trials in bariatric patients and their impact on the development or progression of DS should be acknowledged as vital (see Figure 2).

## 7. Conclusions

DS is a common complication following BS, resulting from anatomical-functional and hormonal alterations of the GIT. Its classification into EDS and LDS forms facilitates diagnosis and treatment, with the OGTT serving as the diagnostic standard.

Dietary interventions remain the first-line therapy. When these fail, various pharmacological strategies—including alpha-glucosidase inhibitors, diazoxide, SAs, GLP-1 agonists, hormonal modulators, and calcium channel blockers—have shown promising results. In refractory cases, surgical reintervention is considered a last resort option. Nonetheless, there is a need to strengthen the evidence through controlled clinical trials and pathophysiological studies in order to optimise the therapeutic approach to DS and improve patients’ clinical outcomes.

## Data Availability

No new data were created or analysed in this study. Data sharing is not applicable to this article.
